# Stroke survivors' and carers' experiences of nutritional care after stroke: a qualitative study

**DOI:** 10.3389/fstro.2026.1733430

**Published:** 2026-05-08

**Authors:** Alex Lang, Sharon Geva, Nicholas R. Evans, Francesca Cavallerio, Débora Vasconcelos e Sá, Ali Ali, Simon Nichols, Sanjoy K. Deb

**Affiliations:** 1 Centre for Better Living, Anglia Ruskin University, Cambridge, United Kingdom; 2 Department of Clinical Neurosciences, University of Cambridge, Cambridge, United Kingdom; 3 Department of Neurosciences, University of Sheffield, Sheffield, United Kingdom; 4 Combined Community and Acute Care Group, Sheffield Teaching Hospitals NHS Foundation Trust, Sheffield, United Kingdom; 5 Chiron, London, United Kingdom; 6 Centre for Cardiovascular Health, Edinburgh Napier University, Edinburgh, United Kingdom

**Keywords:** carer burden, diet, qualitative research, reflexive thematic analysis, semi-structured interviews, stroke, unmet care needs

## Abstract

**Introduction:**

Impaired nutritional status is commonly reported following stroke and is associated with poor clinical outcomes. More than 60% of stroke survivors in the UK are supported by informal, unpaid carers who commonly report one or more unmet care needs. This qualitative study explores stroke survivors' and informal carers' experiences of nutritional care across the pathway of stroke rehabilitation and recovery.

**Methods:**

Twelve stroke survivors and 12 informal carers were recruited via voluntary stroke organizations. Twelve participants were male. Thirteen were aged 18–34, five were aged 35–54, six were aged 55–74. Ten were of Black British ethnicity, nine White British, two Black other, two Asian and one mixed ethnicity. Median time since stroke was 2 years (range: 4 months to >10 years). Individual, online, semi-structured interviews explored the impact of stroke on nutrition, perceptions of nutritional care received, and suggested improvements. Interviews were transcribed and analyzed using reflexive thematic analysis within a critical realist framework.

**Results:**

Four themes were generated from the data: (1) “overlooked stroke-specific and co-morbid challenges,” recognizing the clinical complexity of post-stroke nutrition (2) “the case for personalized, context-sensitive nutrition,” highlighting the importance of personalized nutrition and consideration of cultural factors (3) “preparedness for discharge home,” which recognizes the importance of the multidisciplinary team in preparing stroke survivors and carers for returning home, (4) “a lack of nutritional information and support,” articulating a strong desire for nutritional information and lack of consistent dietetic support after discharge.

**Conclusion:**

Findings highlight the importance of preparing stroke survivors and carers effectively prior to discharge through improved access to personalized, culturally sensitive nutrition information, and more consistent dietetic support. Six recommendations are made for improved nutritional care across the stroke pathway. Strategies are needed to identify and address unmet need in relation to post-stroke nutrition and support recovery of stroke survivors.

## Introduction

Improvements in the organization of stroke care (Morris et al., 2019), and rising stroke incidence due to an aging population (King et al., 2020), mean more individuals and their families are living with the effects of stroke. Stroke is a leading cause of death and adult disability (Feigin et al., 2024), with reports suggesting more than 60% of stroke survivors rely on the help and support of an informal, unpaid carer to help them with day-to-day living, most commonly a spouse or child (The Stroke Association, 2018). Unfortunately, carer burden is frequently reported by those caring for stroke survivors (Kruithof et al., 2016; Pont et al., 2020; Jaracz et al., 2024; Rigby et al., 2009), often arising from the increasing demands placed on carers, which they may feel ill-equipped to meet (Andrew et al., 2015). Carer factors such as increasing age, anxiety, high stroke survivor dependency, and limited family support have been linked to higher carer burden (McCullagh et al., 2005). Furthermore, higher carer burden is also associated with the number of unmet care needs (Andrew et al., 2015) reported by informal carers, and those they care for (Chen et al., 2019; Denham et al., 2022; Zawawi et al., 2020), 73% of whom report at least one long-term unmet need (Chen et al., 2019). These unmet needs for carers are diverse, and include access to professional, psychological, financial, and community support networks; the ability of carers to manage their own emotional wellbeing; and provision of education and advice around rehabilitation, secondary prevention, and lifestyle after stroke (Denham et al., 2022; Zawawi et al., 2020; Tsai et al., 2015). Stroke survivors also report diverse unmet needs including health-related needs, support with community re-integration, and information needs (Zawawi et al., 2020). Unmet care needs are particularly elevated among stroke survivors with disabilities, individuals from ethnic minority groups, and those living in the most deprived areas (McKevitt et al., 2011).

One such unmet need related to life after stroke is diet and nutritional advice, as reported in a systematic review of evidence from survey studies examining unmet needs of stroke survivors (Chen et al., 2019). This included five studies that assessed the prevalence of diet or nutritional advice as an unmet need between 2 months and 8 years following stroke (Brandriet et al., 1994; Groeneveld et al., 2018; Rothwell et al., 2013; Kersten et al., 2002; LoTS Care LUNS Study Team, 2013). Across these studies, a median of 9.3% of stroke survivors (range 4.7–20.9%) reported an unmet need relating to dietary advice, although the specific nature of these nutritional needs was not examined. Another study examining unmet needs at the stroke 6-month review stage in England, reported that both stroke survivors and carers frequently had questions relating to diet (Abrahamson and Wilson, 2019). Furthermore, a UK-wide national survey of 11,000 stroke survivors and carers reported that 21% had not received enough information on diet following their stroke (The Stroke Association, 2018). The limited focus on feeding-related aspects of care in stroke rehabilitation was further highlighted in a study of 206 stroke survivors in the UK, who reported a range of different eating problems after stroke that were often overlooked (Perry and McLaren, 2003). Barriers to eating and drinking following stroke include a combination of physical, psychological and cognitive impairments (Jones and Nasr, 2018), and organizational factors (Jones et al., 2025), which can make optimizing post-stroke nutrition challenging. Malnutrition is prevalent in stroke survivors at all stages of their recovery (Huppertz et al., 2022), and can negatively impact clinical outcomes, increasing mortality, length of hospital stay, and healthcare costs (Gomes et al., 2016). Together, these findings highlight that personalized post-stroke nutrition advice and information remain an important unmet need that warrants further investigation.

Research exploring experiences of nutrition after stroke often focuses on specific aspects or defined timeframes, such as eating and drinking difficulties on the acute stroke unit (Jones et al., 2025), physical feeding difficulties 6 months post-stroke (Perry and McLaren, 2003), dysphagia (Eltringham et al., 2019), and healthy eating (Parappilly et al., 2020). Similarly, studies exploring unmet nutritional need typically focus on specific aspects of care, such as enteral tube feeding (Mou et al., 2022; Mooi and Ncama, 2020). Our study aims to take a broader approach and explore carers' and stroke survivors' experiences of nutritional care across the stroke pathway to identify areas of unmet need and help inform the design and delivery of quality nutritional care for this growing and diverse population. Given the complexity of nutritional care needs following stroke and the limited understanding of how these are experienced and managed by stroke survivors and informal carers across the stroke pathway, a qualitative approach was chosen for its ability to gain a deep understanding of both lived experiences and the meaning that is attached to the experience.

## Methods

### Study design

Individual, online semi-structured interviews and reflexive thematic analysis was used to analyze informal carers' and stroke survivors' experiences of nutritional care across the stroke pathway (Braun and Clarke, 2019). Our study is framed within a critical realist paradigm (Bhaskar, 2013), underpinned by realist ontology—we experience sensations of images and things going on in the real world and not the things directly (Scott, 2005), and relativist epistemology—the diversity of beliefs, perspectives, and interpretations held by individuals or communities (Sayer, 2000). A semi-structured interview technique was selected as it is effective in exploring the experiences, perceptions and opinions of research participants. This approach provided structure to each interview while offering greater flexibility than a structured questionnaire or survey, allowing the interviewer the ability to explore unexpected experiences and perceptions as they arose, and the interviewee to influence the direction of the conversation. Our approach was guided by recommendations for designing and conducting semi-structured interviews in healthcare research (DeJonckheere and Vaughn, 2019). An interview guide (see Supplementary material) was developed by members of the research team based on their clinical and academic experience, relevant academic literature, and the National Clinical Guidelines for Stroke (Intercollegiate Stroke Working Party, 2023).

### Positionality

When undertaking qualitative research, it is important to acknowledge one's positionality (Gurr et al., 2024), providing the reader with insight to which the researchers' worldviews and position shape the study design, data analysis, and the conclusions made. Reflexivity, enables ongoing reflection by the research team, acknowledging how their unique experiences shape any assumptions held. We therefore outline the positionality of our interdisciplinary research team early in the paper to effectively equip the reader. For context, the first author (AL) is a Ph.D. Candidate with many years of clinical experience as a stroke specialist dietitian in the UK, working in the acute hospital setting in urban centers. AL led on the design of the interview schedule, with input from the research team leading to refinement and iteration of the questions. It is important to consider how AL's experience as a hospital-based dietitian may have influenced how the study was designed and conducted. For example, the greater focus of questions toward the acute end of the stroke pathway may reflect their acute hospital experience. Similarly, including carers alongside stroke survivors, was a conscious decision to ensure that carer voices were heard, likely influenced by AL's experience of seeing the vital role they play in supporting the nutritional needs of stroke survivors in hospital and in preparation for home. Positionality is also influenced by the power dynamics between the researcher and participant (Dhillon and Thomas, 2019). AL has not worked clinically with any of the stroke survivors or participants recruited to the study. Participants were recognized and treated as the “expert” during interviews, bringing their wealth of knowledge and experience to the interviews. SG is a cognitive neuroscientist, with an interest in language and memory across the lifespan, particularly following brain damage. NE and AA are Consultants in Stroke Medicine and researchers in Cerebrovascular Disease, providing clinical insights into the research project. FC and DA have expertise in psychology with specific experience in qualitative research methods. SN is an academic healthcare researcher focused on cardiovascular rehabilitation. He also leads research for a digital health company. His work integrates clinical insights with patient-centered innovation. SD is an academic and researcher specializing in nutrition and exercise, with a specialist interest in nutrition after stroke. He is of South Asian background and a proponent of cultural diversity in research and practice, offering a perspective on how the data will be analyzed and interpreted. Researcher self-reflexivity played an important role the study. Regular self-reflection, sense-checking of ideas and discussing interpretations or assumptions of the data with members of the research team were regularly undertaken at each stage of the study, from the design to the interpretation of findings.

### Ethical considerations

The study obtained ethical approval from the relevant Faculty Research Ethics Panel at Anglia Ruskin University (Ref: ETH2324-7167) on 10th July 2024 prior to data collection. A participant information sheet outlining the purpose and details of the study was provided. Participants were made aware that participation was completely voluntary and that findings would be pseudo-anonymised, meaning that identifiable information would be removed from the data, and a code would be assigned instead. Written consent was obtained from participants prior to their interview, and they were informed they could withdraw their consent if they wished. As the interviews covered sensitive topics which had the potential to be upsetting for participants, they were provided with support and signposting to organizations that could offer support if required. Participants were not obligated to answer any questions they didn't wish to, could take their time, have breaks, or leave the session if needed. An aphasia accessible version of the participant information sheet was also available for participants with communication impairment.

### Participants and recruitment

The target population for this study were (1) stroke survivors aged 18 years and older, who received acute stroke care in the UK; and (2) informal carers aged 18 years and older who currently provide, or who have previously provided, unpaid care to a stroke survivor living at home in the UK, and who recognize themselves as a principal carer. Twenty-four participants, comprising 12 stroke survivors and 12 carers who were unrelated to each other, were recruited via one of three different routes: (1) UK-based stroke charities, (2) public engagement “meet the researcher” events the Anglian Stroke Partnership for Increasing Research Engagement (ASPIRE), or (3) the National Institute of Healthcare Research (NIHR) People in Research website (www.peopleinresearch.org). The sample size was selected through the concept of information power for the aims of the study and the sample (Malterud et al., 2016). Twelve participants were male. Thirteen were aged 18-34, five were aged 35–54, six were aged 55–74. Ten were of Black British ethnicity, nine White British, two Black other, two Asian and one mixed ethnicity. Median time since stroke was 2 years (range: 4 months to >10 years). The following nomenclature is used to help the reader understand the characteristics of participants quoted in the results: SS = stroke survivor, IC = informal carer, Ethnicity: WB = White British, BB = Black British, BA = British Asian, AO = Asian other, ME = mixed ethnicity. Gender: F = female, M = male. Age: 18–24, 25–34, 35–44, 45–54, 55–64, 65–74.

### Data collection

Twenty-four semi-structured, online interviews were conducted between September 2024 and March 2025. Informal carer interviews lasted between 25 and 51 min (median 37 min), while stroke survivor interviews last between 21 and 50 min (median of 34 min). To support greater inclusion, participants were given the option of face-to-face or online interviews. All participants opted to undertake interviews virtually using MS Teams. Sociodemographic information (age, sex, ethnicity, type and date of the stroke, carer relationship) was collected from each participant prior to their interview. Interviews were recorded and transcribed verbatim by MS Teams, during which only AL and the participant were present, except for one participant, whose mother was also present for support. Each participant was interviewed once, although some participants met with AL on MS Teams prior to their interview to assess suitability for the study and address any queries. Participants were given a £50 shopping voucher for their participation. Online interviews allowed participation from across the country, capturing experiences from participants treated in different hospitals and with varying access to follow-up care and charitable organizations support.

### Data analysis

A reflexive thematic analysis approach was adopted to generate themes (Braun and Clarke, 2019), allowing for deep engagement with the data. Transcriptions from each interview were reviewed and corrected following the interview by AL. Each transcript was anonymised, with participants given a pseudonym. All data extracts from individual transcripts were preliminarily coded by AL. Transcripts and codes were then independently reviewed and cross-checked by a second researcher (SD), enabling triangulation to reduce the risk of researcher bias. Codes were then arranged into preliminary themes and iteratively refined where necessary. Themes were generated and discussed among the research team, along with a stroke survivor and carer patient and public engagement group. This led to further refinement of theme names and the generation of additional sub-themes.

## Results

**Figure 1:**
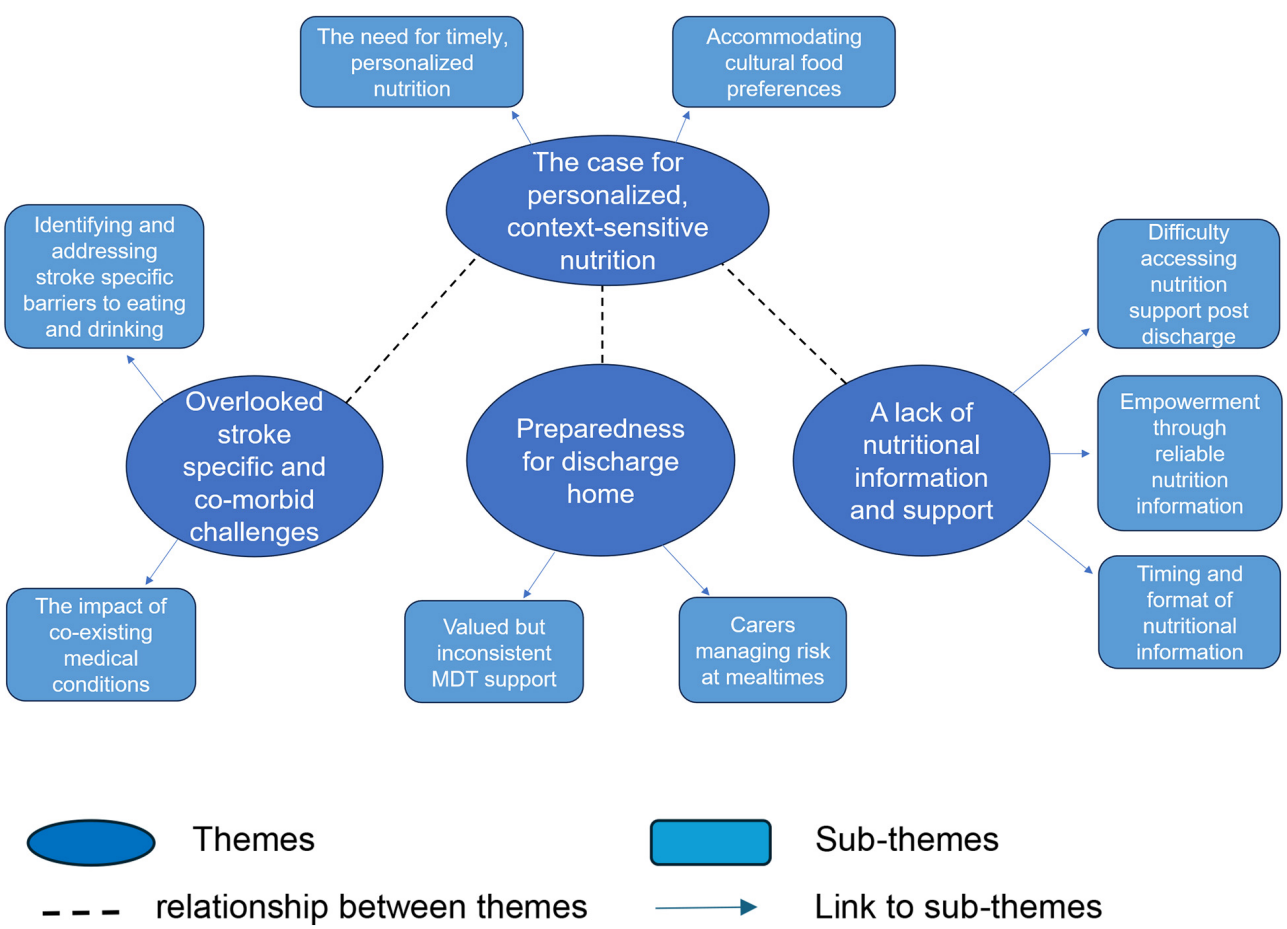
Thematic map outlining the nutritional experiences of stroke survivors and informal carers.

Four key themes were identified through the reflexive thematic analysis (see Figure 1 for the thematic map). Two themes relate to experiences of nutritional care in hospital: “overlooked stroke-specific and co-morbid challenges' and “the case for personalized, context-sensitive nutrition.” A third theme: “preparedness for discharge home” relates to experiences during the transition from hospital to home, while a fourth theme: “a lack of nutritional information and support” relates to experiences of nutritional care in the community. Together, these themes capture the experiences of nutritional care of stroke survivors and informal carers throughout different stages of their rehabilitation and recovery process, the challenges encountered, enablers of care, and unmet needs. Although our results present these as a linear journey of experiences, we acknowledge that this is rarely the case and that themes may occur and reoccur for individuals during different stages and settings of their journey, maintaining their relevance many years after their stroke.

### Overlooked stroke-specific and co-morbid challenges

This theme recognizes the clinical complexity of post-stroke nutrition and comprises two sub-themes (1) identifying and addressing stroke-specific barriers to eating and drinking; and (2) the impact of co-existing medical conditions. It highlights the complexities in addressing the nutritional needs of stroke survivors.

### Identifying and addressing stroke-specific barriers to eating and drinking

A comprehensive nutritional assessment is important for identifying barriers to eating and drinking and supports the development of a personalized nutrition care plan tailored for each stroke survivor. Barriers to eating and drinking caused by cognitive and psychological consequences of stroke were felt to be under-recognized compared to physical impairments, leading to reduced oral intake and associated weight loss:

“*Maybe you know just a bit more on the ball with asking about nutrition, I don't think it's the thing that is at the front of their mind. I think they're more concerned about the physical problems after a stroke. I think that's the same with the psychological side as well, unfortunately” SS11 (WB/F/55-64)*

“*I think that what makes the weight to go then I think he was affected mentally…he was having severe mental health challenges” IC12 (BB/M/25-34)*

This is a complex bidirectional relationship as weight loss and poor nutritional status may worsen mental health, further increasing the risk of malnutrition and poor rehabilitation outcomes. Communication impairment following stroke added a further layer of complexity, particularly when it prevented survivors from expressing food preferences. One stroke survivor outlined how she was eventually offered pictures to overcome this challenge, highlighting the importance of access to adaptive communication tools:

“*I didn't understand words, so eventually one of them caught on and gave me pictures of my food and pointed out I'd like a potato or lasagne…so the pictures really helped me understand the food” SS9 (WB/F/35-44)*

These experiences suggest that timely identification of psychological disorders, communication, and cognitive impairment as potential nutritional barriers form important, but often overlooked components of a personalized nutrition approach. Processes are required that identify these barriers early and lead to practical tailored nutritional interventions. This is especially important for stroke survivors who may not have a carer to advocate for them.

#### The impact of co-existing medical conditions

Co-existing medical conditions adds further complexity in meeting the nutritional needs of stroke survivors. Effective management of co-existing chronic conditions, such as diabetes, became more difficult to manage following acute stroke. Changes in daily routine and meal provision can lead to reduced autonomy impacting ability to balance food alongside timing of multiple medications or insulin. For example, one carer recalled how managing diabetes alongside her grandfather's stroke was a struggle, complicating his nutritional goals:

“*This played a significant impact on his appetite because my grandad… had that type 2 diabetes...so I think it's really a concern for us to also look out for his weight because we're struggling with 2 diagnosis” IC10 (BB/M/25-34)*

Another carer reported that eating and drinking were impacted due to the burden of taking numerous medications for multiple conditions:

“*I believe a lot of factors came into place and 20 plus medications didn't help either” IC6 (AO/F/25-34)*

Stroke survivors needed to advocate for themselves, especially those with co-existing conditions and receiving care under multiple specialities. Frustrations can surface where there is a perceived lack of communication between teams and conflicting advice on what to eat:

“*Think my teams were struggling because I had my cancer and my stroke that they were kind of almost putting me on the next team. So, I'll go to the cancer people, they'll say go to your stroke team. The stroke team will go too and then you get no results. So, I'd have to keep pushing it saying can someone talk to me about something specific” SS9 (WB/F/35-44)*

Addressing these gaps may require developing pathways that prioritize nutrition across conditions, embedding nutrition expertise within multi-disciplinary teams, and ensuring that patients are not left to negotiate conflicting advice on their own. Factors that impact micronutrient intake, absorption and utilization should also be considered as part of a tailored assessment and monitoring plan. One stroke survivor outlined how she was diagnosed with vitamin D and iron deficiencies following her stroke:

“*They discovered that I've got a huge vitamin D deficiency…and also a huge iron deficiency” SS8 (WB/F/55-64)*

Although routine screening for micronutrient deficiency in stroke survivors is not recommended, supplementation for those at high risk of deficiency such as vitamin D in housebound individuals, is advised (Intercollegiate Stroke Working Party, 2023). These experiences provide an important reminder that nutritional considerations after stroke are broader than protein-energy malnutrition alone.

### The case for personalized, context-sensitive nutrition

This theme highlights the importance of tailoring strategies to the individual based on identity, culture, and personal preference and is comprised of two sub themes: (1) the need for timely, personalized nutrition; and (2) accommodating cultural food preferences. Here, “context” refers to the individual's cultural and lived experience rather than their position within the care pathway. Together, these findings emphasize the need for individualized, culturally competent nutritional support, particularly in the early stages of care, to improve clinical outcomes and quality of life after stroke.

#### The need for timely, personalized nutrition

Due to the heterogeneity of impairment following stroke, carers emphasized that tailoring nutritional care to the individual was an important consideration:

“*We had been given a more personalised nutrition based on the individual needs of my father - his preferences and I found this to be so beneficial” IC1 (BB/M/18-24)*

As stroke survivors have different nutritional needs based on their weight and clinical condition, a personalized nutrition approach appears necessary to optimize nutritional status and support rehabilitation. Comparisons were made between generic and personalized support, with stroke survivors expressing the increased benefit and appreciation for tailored interventions when compared to a more generic approach.

“*I think if you could personalise it, it would make such a difference” SS9 (WB/F/35-44)*

However, this personalization was seldom received, with stroke survivors predominantly sharing their disappointment and desiring a personalized, bespoke approach to the nutritional advice they received rather than the generic “one size fits all” approach most experienced:

*It was very generic…I wasn't given a dietitian” SS6 (BA/M/25-34)*

“*…it's rather one size fits all…so they were just giving me nutritional handouts that they had…There was no individual questions asked…on a personal basis” SS11 (WB/F/55-64)*

Notably, stroke survivors identified the first month to be a critical window for nutrition support, emphasizing significant weight loss during this period when nutritional intake was compromised:

“*I think someone said it was 2 stone within a couple of weeks* (weight loss) *because I remember looking at my body for the first time and thinking, Oh my God, it's like this is crazy. I've never seen myself like this since I was younger” SS9 (WB/F/35-44)*

These insights highlight the importance of prompt nutritional screening and timely, personalized nutritional interventions in meeting the unique needs of stroke survivors and reducing the emotional burden of informal carers.

#### Accommodating cultural food preferences

Cultural factors form a crucial component of a personalized nutrition approach. Stroke survivors and carers from diverse ethnicities and cultures consistently emphasized the need for culturally appropriate meals and tailored nutrition guidance. However, respondents felt that the meals provided in hospital failed to reflect their cultural preferences, leading to reduced food intake and dissatisfaction. Stroke survivors vividly recalled how they felt when their dietary preferences were overlooked on the stroke unit:

“*I'm vegetarian and I'm mostly sort of plant-based vegetarian, and I wasn't really getting the choices and the options…and that made me feel really undermined and quite distressed, actually…made me feel really dehumanised” SS7 (WB/F/25-34)*

Carers were critical of the one-size-fits-all approach in stroke management, advocating instead for greater inclusivity in nutritional care to support recovery:

“*Personalized management is really important. It cannot be over emphasized because I think what's out there most of the sessions we have received, I think it's built more on a generalized form of stroke management that doesn't really have cultural entities in in it” IC10 (BB/M/25-34)*

These insights support that recognizing and accommodating cultural food preferences is essential not only for nutritional adequacy but also for preserving the social and emotional wellbeing of stroke survivors and carers. For those from minority ethnic backgrounds, culturally misaligned food was experienced as an exclusion from care itself. Addressing this requires structural changes to menu planning, procurement processes and workforce training, alongside the inclusion of diverse voices in the development of care pathways.

### Preparedness for discharge home

This theme emphasizes the value, but inconsistency of the multi-disciplinary team (MDT) in preparing carers and stroke survivors for returning home. It explores the transition from hospital to home, focusing on the experience of preparedness for caring for the stroke survivors' nutritional needs once at home. Both stroke survivors and carers highlighted the importance MDT support, practical training, and timely information, yet experiences varied widely, with a smooth transition a rarity and most feeling overwhelmed and ill-equipped.

#### Valued but inconsistent multi-disciplinary team support

Stroke survivors and carers frequently emphasized the critical role of MDT support in preparing them for life following discharge from hospital. The transition from hospital to home is a significant milestone for stroke survivors and informal carers alike, with carers often assuming roles previously provided by the MDT team. Effective MDT support was characterized by structured guidance, hands-on training, and access to specialist professionals. When available, such support significantly improved carer confidence in managing nutritional care at home. One young carer, who felt well-prepared described receiving everything he needed:

“*For me, I think I received everything that I needed at that time to improve the well-being of my dad” IC1 (BB/M/18-24)*

While gratitude to the stroke MDT's input and the significant impact that carer education had on recovery and quality of life was also recognized:

“*I think the education that I got for my caregivers – the physician, the dietitians, everybody like that was there at that point actually helped to support my recovery – actually helped to support my eating and drinking. I would say I owe it. I owe all the appreciation to them at this moment, because when I look back, I realized that it is because of the education” SS1 (BO/F/25-34)*

Other stroke survivors highlighted the importance of access to specialities such as dietetics and speech and language therapy, especially where malnutrition and dysphagia were present:

“*Most of this advice you're giving to me, like when she was working hand in hand with the dietitian. Help to plan the food and fortify nutrients in the food” SS2 (BB/M/25-34)*

However, carers and stroke survivors described significant gaps in training and information that was inadequate or poorly timed. Some carers felt rushed into taking responsibility before they were ready making them feel too overwhelmed to absorb information:

“*The experience was so overwhelming. The doctors and the therapists they tried to give us a lot of information that personally I found so hard to absorb in the middle of such an emotional crisis. But we had to” IC1 (BB/M/18-24)*

“*I was told to persevere…there was no mention of dietitian” SS12 (ME/F/55-64)*

These findings highlight how the variation in MDT support leads to differing feelings of preparedness among stroke survivors and carers. While some benefited from structured guidance and hands-on training, others felt rushed, overwhelmed, or unsupported that resulted in unmet need. Variation in preparedness appeared to reflect differences in local service provision and workforce capacity, suggesting that nutritional preparation for discharge is not consistently embedded within stroke pathways.

#### Carers managing risk at mealtimes

The most significant barrier to eating and drinking was dysphagia, affecting stroke survivors during the acute phase following stroke and, for some, persisting beyond hospital discharge. Carers described the emotional and practical challenges of managing this risk, with one carer summarizing the struggle vividly:

“*It is really difficult swallowing, trying to sit upright, initial stage in the hospital…so coping with eating and drinking during the stroke was so challenging” IC9 (BB/F/25-34)*

The risk of choking and aspiration pneumonia, a common complication of dysphagia, weighed heavily on carers who had the responsibility of preparing meals at home while safely adhering to texture recommendations:

“*It was a careful process, especially after having the hospital staff handle it for so long. I became, yeah. I became more aware of how slow things needed to go. I remember meals took longer, but we had to be patient to make sure that we weren't rushing him because, you know, if at that time you had rushed him, maybe we could have put him at risk of being choked” IC1 (BB/M/18-24)*

By working with the MDT, carers picked up useful practical strategies to manage and reduce the risk of aspiration and choking incidences, helping them feel better equipped and more confident. One carer described strategies she had been taught by the MDT prior to discharge:

“*To always make her sit upright, a kind of a 90*°*, you know, while eating and drinking and for 30 minutes afterwards just to prevent kind of aspiration or choking” IC9 (BB/F/25-34)*

An important aspect of the carer experience is therefore feeling equipped to prepare food and drink safely and to treat potential complications such as choking. This highlights a priority area for personalized MDT discharge guidance.

### A lack of nutritional information and support

This theme articulates a strong desire for reliable nutritional information, leading to self-development and feelings of increased satisfaction, confidence, and empowerment, but also a lack of consistent dietetic support after discharge. While some stroke survivors actively sought and developed their own knowledge, others struggled to access clear, reliable information, leading to stress and unmet needs. The format in which nutritional information was presented and the timing of when it was received were important considerations for maximizing their impact. A lack of consistent dietetic support was also reported after discharge, increasing uncertainty and leading to a greater burden on carers.

#### Difficulty accessing nutritional support post-discharge

Nutritional information and support were easier to access as an inpatient, but became harder after discharge, leading to frustration and disappointment among stroke survivors:

“*Once you come out of hospital, there was no real aftercare…the aftercare was rubbish” SS10 (WB/F/55-64)*

“*I had no nutritional advice whatsoever” SS8 (WB/F/55-64)*

With access to “reliable information” from healthcare professionals harder to access following discharge home, many turned to the internet in the absence of formal guidance, often with anxiety about the quality or credibility of what they found. When information was hard to locate, stroke survivors and carers described feeling stressed, worried and overwhelmed:

“*I did Google searches and things I did not always the best thing to do, but in the absence of anything else…” SS4 (WB/M/65-74)*

“*I think I was quite stressed with it. Also, initially I didn't feel equipped and so I was a bit worried that maybe I was giving him too much or, you know, and things like that” IC5 (WB/F/45-54)*

Where stroke early supported discharge (ESD) teams included professionals whose support addressed specific stroke survivor needs, such as physiotherapy and occupational therapy, their input was seen as hugely beneficial. But the lack of nutritional input from ESD teams was highlighted by those stroke survivors who had ongoing nutritional needs at home, representing an area in need of development within ESD and community rehabilitation teams to optimize rehabilitation outcomes:

“*None of that was food related*. *None of it was swallowing related. It was all physical therapy and that kind of thing. So, I kind of felt like I was left on my own a little bit” SS5 (WB/M/35-44)*

These insights show a clear gap in post-discharge nutritional support, highlighting the need to extend access to specialist dietary advice beyond the acute stroke unit.

#### Empowerment through reliable nutritional information

Access to information was felt to be an important coping strategy as it played a significant role in helping carers feel empowered, which in turn led to improved ability to better manage the condition, reduce carer burden, and improve quality of life. Timely access to support was transformative, enabling carers to feel adequately informed and supported:

“*The more information people have…the better they can understand the role that nutrition has, you know, plays in the recovery process…if you're better informed you feel more in control, more empowered” IC8 (BO/F/45-54)*

The provision of information and training also helps with self-development and the satisfaction that carers feel from the role that they play in the nutritional care of stroke survivors. Increased nutritional knowledge translated into noticeable improvements in eating and drinking:

“*I'm very happy because I feel like I'm doing a good job, in as much as at some point I felt like I didn't know what I was doing…but I was willing to take care of him. But he's responding well. I think his appetite has increased. He is eating well now and the supplements too. It is good. He's eating well and I'm really happy about that” IC3 (BB/M/25-34)*

This captures the emotional journey from feeling under-prepared to being able to apply nutritional knowledge, reflecting a growing confidence with external validation from stroke survivor feedback reinforcing this. It also outlines a link between improved eating in stroke survivors, their recovery and enhanced carer wellbeing.

#### Timing and format of nutritional information

The timing and delivery format of nutritional information significantly influenced how stroke survivors and carers engaged with and applied post-stroke dietary guidance. Access to information provided face to face and over the phone, for example by charitable organizations after discharge from hospital, was highly valued:

“*There was a session and we did have one session which was themed around nutrition for brain injury…they were talking about what kinds of food are particularly good for brain injury recovery” SS7 (WB/F/25-34)*

However, the shift to predominantly remote services following the COVID-19 pandemic elicited mixed responses. While some carers found online formats more convenient and less intimidating, others perceived virtual interactions as unsuitable, particularly for those who struggled to engage digitally:

“*He kind of gave up at that stage because it was all online” IC5 (WB/F/45-54)*

The format and delivery of nutritional information was a crucial factor influencing engagement with post-stroke dietary guidance. Digital tools, particularly those offering video content or app-based support, were identified as more accessible and user-friendly than written materials. Carers framed these tools not only as practical aids but also as mechanisms for enhancing self-efficacy, especially when they included relatable narratives or real-life examples:

“*If there was something like an app where you could have kept information so he could have had videos about real life stories” IC5 (WB/F/44-54)*

Carers also outlined that the financial strain of providing nutritional care at home is an under-recognized challenge. Growing food security challenges in the UK, the wider social context should be considered when providing nutritional guidance. Furthermore, there must be consideration of how to minimize digital exclusion and health inequalities for those who may not have the financial means to participate in digital interventions:

“*Some family maybe having financial burden. You know, it could be quite, very expensive to have from specific nutritional plan” IC4 (BB/M/18-24)*

Overall, stroke survivors and carers called for healthcare support that was multi-modal, based on peer support and lived-experience, accessible via a range of formats, including for those less technologically minded or suffering from digital exclusion. The variation in access to information and resources reported by stroke survivors and carers in this study highlights the significant impact of structural factors, such as health service design and resource allocation, on how nutritional care is experienced across different parts of the country. These accounts also suggest that socioeconomic circumstances interact with health needs to shape nutritional recovery after stroke, with financial constraints influencing the feasibility of recommended dietary changes.

## Discussion

This study explored the experiences of stroke survivors and informal carers managing nutritional needs on the acute stroke unit, peri-discharge and in the community, highlighting the challenges, strategies, and unmet support needs reported across the stroke pathway. The findings suggest that a uniform approach to post-stroke nutrition is ineffective, with stroke survivors and carers alike emphasizing the importance of timely, personalized, and culturally competent nutritional care in hospital. On transition home and in the community, while some carers successfully adapted to their roles through self-learning and trial-and-error, many struggled due to a lack of structured nutritional education, inconsistent access to dietetic support, and the emotional toll of providing care. Stroke-specific barriers such as dysphagia, cognitive impairments, and mental health sequelae further complicated nutritional care, often leaving stroke survivors and carers feeling overwhelmed and unsupported. The absence of ongoing professional nutrition guidance after discharge highlights a significant gap in stroke aftercare, reinforcing the need for accessible, community-based specialist dietetic support and tailored digital resources. Across themes, participants' experiences of nutritional care were shaped not only by individual clinical needs but also by wider structural and social factors. Service organization, workforce capacity, and resource availability influenced access to personalized nutritional support. These findings suggest that improving nutritional care after stroke requires both individualized interventions and system-level changes.

Participants in this study broadly described personalized nutrition as the tailoring of nutritional advice toward themselves based upon their unique personal needs and characteristics. Research suggests such characteristics include genetic and phenotypic traits, pre-existing medical, nutritional, and other relevant information and is grounded in the belief that bespoke nutritional advice is more effective than a generic approach (Ordovas et al., 2018). Evidence supports benefits of a personalized nutrition approach in improving healthy eating habits and cardiovascular risk factors (Celis-Morales et al., 2016; Cross et al., 2025). Furthermore, frequent and individualized nutrition advice is also associated with improved nutritional status, physical function, and dysphagia after stroke (Shimazu et al., 2021). Improved post-stroke outcomes were reported in trials where individuals received nutritional interventions tailored to their individual nutritional requirements (Otsuki et al., 2020; Ha et al., 2010). This was echoed in the experiences of the stroke survivors and cares in the current study who articulated a clear preference for individualized nutritional care, viewing it as more relevant, acceptable, and supportive of recovery than standardized advice. This preference appeared to stem not only from perceived physical benefits, but also from its emotional and psychological effects, including a sense of being valued and understood within the care process.

Another important finding comes from experiences of the transition from hospital to home, a particularly important stage in the stroke survivors and carer journey. Abrahamson and Wilson (2019) identified “hotspots” in the care pathway where patients and carers felt particularly unsupported. These typically occur during periods of transition, such as from hospital to home (Chen et al., 2021), and was something stroke survivors and carers experienced in this study. While participants in our study described the positive impact of dietetics, and speech and language support for dysphagia management, studies recognize the important role that the wider MDT play in addressing the nutritional needs of stroke survivors (Jones and Nasr, 2018; Perry et al., 2013). For example, training carers in basic nursing and personal care techniques during stroke rehabilitation, including guidance on nutrition, reduces carer burden (Kalra et al., 2004). Current clinical guidelines outline that carers should be offered an educational programme covering various aspects of stroke care in preparation for home (Intercollegiate Stroke Working Party, 2023), with strategies that actively involve carers and stroke survivors appearing to be beneficial, improving carer quality of life and reducing anxiety and depression (Crocker et al., 2021). The extent of this provision in clinical practice is inconsistent, given that 85% of carers in a national survey reported they had not received the support and information they needed to equip them for their caring role (The Stroke Association, 2018). This study provides context for these figures, highlighting the need to engage carers early and throughout the discharge process. Enabling carers to support the stroke survivor on the ward under supervision of the MDT can be a useful way of building their skills and confidence in a supportive environment (Hekmatpou et al., 2019).

Participants in this study described how access to reliable information during the transition home and in the community fostered a sense of empowerment, enabling greater self-efficacy and confidence in managing nutritional care. This is supported by findings from a randomized controlled trial showing that providing education through an online training programme, using WhatsApp as the mode of delivery, led to improvements in the quality of life for stroke survivors and a reduction in the care burden for their carers (Nazari et al., 2024). The mode of delivery of the 15-session programme which included a session on nutritional care, is aligned with findings from our study—where digital tools, particularly those offering video content or app-based support, were identified as more accessible and user-friendly than written materials. Access to information can lead to greater self-advocacy and active participation in care, allowing individuals to express their wishes and take more ownership of their health. Taken together, this evidence suggests that tailored information and support can lead to elevated levels of empowerment and reduced carer burden. However, in our study participants described a significant unmet need and frustrations accessing reliable nutritional information and timely support. This led them to rely on the internet for nutritional information, raising concerns about the reliability of the information they obtained. Research demonstrates that individuals often look beyond the health and social care system for sources of information and support (Forster et al., 2021).

Participants described the value of information and support provided by trusted stroke charities, digital tools (such as apps), and peers to support improved nutritional care at home. Self-management interventions after stroke have been found to improve quality of life and self-efficacy (Fryer et al., 2016), while peer-support has been identified as a way of addressing unmet needs among stroke survivors (Corbin et al., 2023). Peer support is highly valued by stroke survivors and has been identified as a key component of effective self-management interventions, facilitating knowledge exchange and collaborative problem solving between peers (Clark et al., 2020). A study on the effect of peer education on stroke prevention (Kronish et al., 2014) in an urban minority ethnic population of stroke survivors found that a 6-week, peer-led, community-based self-management workshop focusing on stroke prevention significantly increased the number of participants achieving blood pressure targets compared to a waiting list control group. Although there were no group differences in cholesterol control or use of antithrombotic medications, the authors noted that a peer-led approach may be helpful in reducing disparities in stroke outcomes. In another study, peer support via text message intervention to promote physical activity in stroke survivors was perceived as beneficial beyond physical activity, with participants also reporting feeling more supported and connected (Farre et al., 2023). Text messages included peer support in the form of quotes from other stroke survivors offering encouragement and support. These studies reinforce peer support as an important enabler for self-management after stroke.

Digital approaches may offer promising opportunities to improve access to nutritional information and support following stroke. Smartphone-based interventions have been shown to improve lifestyle risk factors among stroke survivors, with participants generally reporting positive attitudes toward their use, particularly when digital tools supplement rather than replace clinical care (Frühwirth, 2020). However, there is a lack of studies exploring digital interventions to improve dietary behaviors among stroke survivors. In non-clinical populations, a recent systematic review of 52 studies by Vanwinkelen et al. (2026) demonstrated a small but significant positive effect of digital interventions (including SMS text messaging, web-based, social media, and mobile app interventions) on dietary intake, although findings varied by population and intervention type. Interventions included a range of behavior change techniques, and those delivered via social media or SMS text messages had the greatest effect, potentially highlighting the important role that peer support plays in effective dietary change. The unmet need for digital tools to improve nutritional knowledge and support, as described by participants in our study, alongside opportunities for increased digital peer support in the stroke population, warrant further investigation.

The variation in access to nutritional care observed in this study could be due to structural factors inherent in the design and delivery of stroke care in the UK. Despite significant improvements in the organization and delivery of acute stroke care (Morris et al., 2019), wide socioeconomic disparities exist in the burden of stroke in England (Bray et al., 2018). Underserved and minority populations may be at greater risk of complicated care transitions which in turn, can negatively impact stroke recovery (Reeves et al., 2023). Significant variation in access to resources also exist, with the recent annual report from the Sentinel Stroke National Audit Programme (SSNAP) reporting that 78% of patients identified at risk of malnutrition were assessed by a dietitian prior to hospital discharge from the acute stroke unit (Sentinel Stroke National Audit Programme, n.d.). The range reported—from 21% to 100% of eligible patients being assessed by a dietitian—highlights significant variation and an inequity in access to specialist nutrition assessment between stroke units. As dietetic care in hospital is an influential predictor of post-hospital nutritional care (Keller et al., 2018), access to dietitians on the acute stroke unit is critical for continuity of nutritional care and support in the community. Additionally, while recommended resourcing levels for dietetics exists within the acute stroke MDT, the role is not recognized in ESD or community rehabilitation teams resourcing (Intercollegiate Stroke Working Party, 2023), despite evidence of impaired nutritional status in community-dwelling stroke survivors (Huppertz et al., 2022). Reports of participants receiving generic information may suggest structural constraints within stroke services, where limited dietetic capacity and standardized educational materials may restrict opportunities for individualized nutritional support.

A strength of this study is it explored the experiences of a diverse group of individuals affected by stroke across the care pathway, including both stroke survivors and carers. Over half of participants were aged under 35 years old while nearly two thirds of participants were of Black, Asian or mixed ethnicity. There were more male carers interviewed than female carers. While this sample is not necessarily representative of the UK stroke population, it allowed us to explore the experiences of groups of stroke survivors and carers who are often underrepresented in UK medical research (Smart and Harrison, 2017). The ethnic characteristics of the sample may explain the sub-theme on accommodating cultural food preferences, which represents a key component of a personalized nutrition approach. Exploring the experiences of young stroke survivors and carers is important as the incidence of stroke is rising in younger adults of working age (Nehme and Li, 2026). Furthermore, with evidence suggesting that minority ethnic groups have earlier onset of stroke by about 5 years compared to Caucasian individuals (Fluck et al., 2023), there may be a disproportionate burden placed upon younger carers, and carers from minority ethnic families. This further highlights the value in understanding the experiences of a diverse group of individuals affected by stroke for developing more inclusive and effective nutritional strategies.

### Implications for clinical practice

Findings from this qualitative study highlight that nutrition matters to stroke survivors and carers. This reinforces the importance of a stroke MDT that is nutrition aware, with effective processes in place that enable timely identification of stroke survivors at nutritional risk, timely access to personalized nutritional interventions, and monitoring across the stroke pathway. Based on these experiences, we outline six practice recommendations that may be helpful for those seeking to address the unmet nutritional needs described by stroke survivors and their carers (Table 1). Education and training are feasible and effective methods for enhancing knowledge and clinical skills within the acute stroke MDT, resulting in improved clinical care and patient satisfaction (Zhao et al., 2024). Our findings suggest that topics such as nutritional screening, the identification and management of barriers to eating and drinking and raising awareness of cultural menu options would be beneficial. Supportive management is an important enabler for a successful education and training programme for the MDT and could be delivered within existing processes, such as local induction training supported by clinical competencies. Similarly, swallowing and nutritional components of a structured training programme that prepares carers and stroke survivors for discharge from hospital would be beneficial alongside other aspects of the caregiving role. This should be supported by information available in a range of formats that cater for diverse communication and cognitive needs. As demand for stroke unit beds increases and the direction of NHS care moves from “hospital to home” (Gov.UK, 2025), access to dietetic resource in stroke ESD and community rehabilitation teams would support equitable access to the personalized nutrition that stroke survivors and their carers crave. Evidence is required to support such policy recommendations and consensus amongst the clinical guideline development group. Furthermore, including a question on nutritional status at the 6-month review stage, with prompts for dietetic referral and signposting to information, could further address an important unmet need.

**Table 1:** Practice recommendations for improved post-stroke nutritional care.

Recommendation	Action
In hospital	
1. Embed a personalized nutrition approach as a core component of high-quality stroke care	– Assess nutritional risk and individual needs early, including medical, functional, cultural, and psychological factors. – Ensure visible and consistent inclusion of dietetic expertise within stroke MDTs. – Tailor recommendations to the individual's unique characteristics, avoiding “one-size-fits-all” advice in favor of nuanced, context-aware guidance. – Offer culturally appropriate meal and snack options that respect individual preferences.
In preparation for discharge	
2. Ensure nutrition is given equal priority alongside other essential aspects of stroke care during the transition between care settings	– Start carer training and education early in the inpatient stay, allowing for practice under supervision. – Deliver structured, practical training complemented by accessible resources for ongoing reference. – Actively involve carers in discharge planning and decision-making as recognized partners in care.
3. Equip carers with the knowledge and skills to confidently manage mealtime risks	– Consistently train carers in dysphagia management: safe food and drink preparation, positioning, pacing, recognizing choking and aspiration signs. – Supply clear, accessible written and/or video materials to reinforce learning and support care at home. – Follow up post-discharge to address ongoing concerns and build confidence.
4. Ensure timely access to reliable nutritional information post-discharge	– Develop and distribute clear, evidence-based resources tailored to nutritional needs after stroke. – Provide a list of trusted websites, charities, and helplines to empower, and reduce reliance on unreliable sources. – Facilitate equitable access to dietetic professionals in the community.
In the community	
5. Leverage digital tools and peer-support networks	– Develop or recommend interactive, trustworthy digital tools (apps, videos) to complement nutritional care. – Ensure digital resources are accessible to users with low digital literacy, communication, or cognitive impairments. – Encourage or facilitate peer-support groups (online or in-person) to share lived experience.
6. Identify and address structural barriers and health inequities that impact access to quality nutritional care	– Ensure visible and consistent inclusion of dietetic expertise within stroke MDTs (including ESD, and community-rehab teams). – Include a nutrition prompt in the 6-month review proforma. – Provide culturally sensitive nutritional care that actively involves individuals from minority ethnic groups, fostering a sense of dignity and respect.

Although many of these recommendations may be nothing new to members of the stroke MDT, implementation can be challenging. Considering the role that the wider stroke community can play could offer a novel approach to improving nutritional care. For example, in some areas, charitable organizations undertake 6-month reviews, providing more time to complete the reviews and offering extra delivery methods than acute NHS trusts (Holmes et al., 2025). They also provide in-reach services to acute stroke units, offering support, signposting and continuity for stroke survivors during the transition home. Charitable organizations also publish valued nutritional resources on their websites, provide local nutrition education sessions, and peer support for stroke survivors and carers. Further collaboration with charitable organizations to improve nutritional care across the stroke pathway would be an promising opportunity to explore. The desire for access to digital tools for improved nutritional knowledge and peer support aligns with the NHS 10-Year Health Plan (2025), which outlines a strategic shift “from analog to digital” (Gov.UK, 2025). The increased use of the NHS App proposed in the plan could make it the go to technology for stroke survivors and carers seeking trusted information and support while also reducing the variation in access across the country. In summary, consideration should be given to how to foster a culture of high-quality, safe, and compassionate nutritional care across the stroke pathway, supporting a uniform approach geographically that is achievable and realistic within the existing care pathways, workforce resources, and financial budgets available. While we acknowledge this may be challenging to implement, we hope that the experiences reported in this study provide food for thought for those delivering and organizing stroke care in the UK and beyond.

### Limitations

It was notable that all interviews were undertaken virtually, a choice which may be explained by the disability of some stroke survivors, carer responsibilities, and the geographical spread of participants across the UK, which made face-to-face interviews more challenging. As participants were recruited via email or online adverts, and interviews were conducted online, participants who were unable to travel or were unconfident with digital technology, or who struggled to effectively communicate online, may have felt excluded. These factors may help explain the relatively young cohort in our study and which may therefore have implications for the transferability of some findings to non-working age stroke survivors and carers. For example, the desire for digital solutions expressed by some younger participants should be balanced against the views of those who are less confident with technology to ensure a variety of solutions that cater for different needs. Additionally, the experiences of non-English speaking stroke survivors and carers, or those with severe communication or cognitive impairments, were not captured, which limits the transferability of our findings to these populations. Future research should be designed with consideration of which methods are most effective to support understanding of their experiences. This is especially relevant as stroke survivors with severe communication or cognitive impairments may be at greater risk of nutritional issues, more dependent on carers, and require provision of information in adapted formats that were not captured in this study. Similarly, as stroke survivors with limited English proficiency can face barriers to equitable care across the stroke pathway (Clark et al., 2022), studies using interpreters or involving researchers from similar backgrounds could support increased awareness of the ingrained cultural challenges this population faces regarding nutritional care. The recruitment methods used in this study introduce self-selection bias, as participants who proactively seek out research opportunities and charitable support may not be representative of the wider stroke survivor and carer community. Participants were recruited at differing stages of their recovery. While this means that recollections of the acute experience may vary, it captures longer-term experiences and highlights that unmet nutritional care needs can persist for many years after a stroke. Despite participants being an average of 2 years since the stroke, our findings had a greater focus toward nutritional care received in the hospital setting. This is an interesting finding that may be due to the lasting impact the hospital experience has had on participants, or to the way the study was designed and analyzed by the research team, based on their experience and beliefs.

### Conclusion

This qualitative study provides unique insights into the experience of stroke survivors and carers, the challenges faced, and strategies employed in providing nutritional care across the stroke pathway. The findings highlight the importance of effectively preparing carers and stroke survivors prior to hospital discharge through improved access to personalized, culturally sensitive nutrition information, and more consistent access to information and support in the community. Informed by these findings, six recommendations are made for improved nutritional care following stroke (Table 1). A longitudinal qualitative research approach could further help understand how the nutritional needs of stroke survivors and carers evolve over time. Strategies are needed for advancing peer-support systems, and the co-design and adoption of resources and digital health tools, that address the unmet nutritional needs reported by stroke survivors and their carers.

## Data Availability

The raw data supporting the conclusions of this article will be made available by the authors, without undue reservation.

## References

[B1] Abrahamson V. Wilson P. M. (2019). How unmet are unmet needs post-stroke? A policy analysis of the six-month review. BMC Health Serv. Res. 19:480. doi: 10.1186/s12913-019-4210-2 31299952 PMC6624961

[B2] Andrew N. E. Kilkenny M. F. Naylor R. Purvis T. Cadilhac D. A. (2015). The relationship between caregiver impacts and the unmet needs of survivors of stroke. Patient Prefer. Adherence 9, 1065–1073. doi: 10.2147/PPA.S85147 26251579 PMC4524576

[B3] Bhaskar R. A. (2013). Realist Theory of Science. London: Routledge. doi: 10.4324/9780203090732

[B4] Brandriet L. M. Lyons M. Bentley J. (1994). Perceived needs of poststroke elders following termination of home health services. Nurs. Health Care 15, 514–520. 7731562

[B5] Braun V. Clarke V. (2019). Reflecting on reflexive thematic analysis. Qual. Res. Sport Exerc. Health 11, 589–597. doi: 10.1080/2159676X.2019.1628806

[B6] Bray B. D. Paley L. Hoffman A. James M. Gompertz P. Wolfe C. D. A. . (2018). Socioeconomic disparities in first stroke incidence, quality of care, and survival: a nationwide registry-based cohort study of 44 million adults in England. Lancet Public Health 3, e185–e193. doi: 10.1016/S2468-2667(18)30030-6 29550372 PMC5887080

[B7] Celis-Morales C. Livingstone K. M. Marsaux C. F. M. Macready A. L. Fallaize R. O'Donovan C. B. . (2016). Effect of personalized nutrition on health-related behaviour change: evidence from the Food4me European randomized controlled trial. Int. J. Epidemiol. 45:dyw186. doi: 10.1093/ije/dyw186 27524815

[B8] Chen L. Xiao L. D. Chamberlain D. Newman P. (2021). Enablers and barriers in hospital-to-home transitional care for stroke survivors and caregivers: a systematic review. J. Clin. Nurs. 30, 2786–2807. doi: 10.1111/jocn.15807 33872424

[B9] Chen T. Zhang B. Deng Y. Fan J.-C. Zhang L. Song F. . (2019). Long-term unmet needs after stroke: systematic review of evidence from survey studies. BMJ Open 9:e028137. doi: 10.1136/bmjopen-2018-028137 31110106 PMC6530326

[B10] Clark E. MacCrosain A. Ward N. S. Jones F. (2020). The key features and role of peer support within group self-management interventions for stroke? A systematic review. Disabil. Rehabil. 42, 307–316. doi: 10.1080/09638288.2018.1498544 30325686

[B11] Clark J. R. Shlobin N. A. Batra A. Liotta E. M. (2022). The relationship between limited english proficiency and outcomes in stroke prevention, management, and rehabilitation: a systematic review. Front. Neurol. 13:790553. doi: 10.3389/fneur.2022.790553 35185760 PMC8850381

[B12] Corbin S. Damiolini E. Termoz A. Huchon L. Rode G. Schott A.-M. . (2023). Rehabilitation professionals' views on individual peer support interventions for assisting stroke survivors with reintegration into the community: a qualitative study. Disabil. Rehabil. 45, 4413–4423. doi: 10.1080/09638288.2022.2152115 36576210

[B13] Crocker T. F. Brown L. Lam N. Wray F. Knapp P. Forster A. . (2021). Information provision for stroke survivors and their carers. Cochrane Database Syst. Rev. 11:CD001919. doi: 10.1002/14651858.CD001919.pub4 34813082 PMC8610078

[B14] Cross V. Stanford J. Gómez-Martín M. Collins C. E. Robertson S. Clarke E. D. . (2025). Do personalized nutrition interventions improve dietary intake and risk factors in adults with elevated cardiovascular disease risk factors? A systematic review and meta-analysis of randomized controlled trials. Nutr. Rev. 83, e1709–e1721. doi: 10.1093/nutrit/nuae149 39420556 PMC12166176

[B15] DeJonckheere M. Vaughn L. M. (2019). Semistructured interviewing in primary care research: a balance of relationship and rigour. Fam. Med. Commun. Health 7:e000057. doi: 10.1136/fmch-2018-000057 32148704 PMC6910737

[B16] Denham A. M. J. Wynne O. Baker A. L. Spratt N. J. Loh M. Turner A. . (2022). The long-term unmet needs of informal carers of stroke survivors at home: a systematic review of qualitative and quantitative studies. Disabil. Rehabil. 44, 1–12. doi: 10.1080/09638288.2020.1756470 32393074

[B17] Dhillon J. K. Thomas N. (2019). Ethics of engagement and insider-outsider perspectives: issues and dilemmas in cross-cultural interpretation. Int. J. Res. Method Educ. 42, 442–453. doi: 10.1080/1743727X.2018.1533939

[B18] Eltringham S. A. Pownall S. Bray B. Smith C. J. Piercy L. Sage K. . (2019). Experiences of dysphagia after stroke: an interview study of stroke survivors and their informal caregivers. Geriatrics 4:67. doi: 10.3390/geriatrics4040067 31817883 PMC6960615

[B19] Farre A. Morris J. H. Irvine L. Dombrowski S. U. Breckenridge J. P. Ozakinci G. . (2023). Exploring the views and experiences of people recovering from a stroke about a new text message intervention to promote physical activity after rehabilitation-keeping active with texting after stroke: a qualitative study. Health Expect. 26, 2013–2022. doi: 10.1111/hex.13809 37409460 PMC10485328

[B20] Feigin V. L. Abate M. D. Abate Y. H. Abd ElHafeez S. Abd-Allah F. Abdelalim A. . (2024). Global, regional, and national burden of stroke and its risk factors, 1990–2021: a systematic analysis for the Global Burden of Disease Study 2021. Lancet Neurol. 23, 973–1003. doi: 10.1016/S1474-4422(24)00369-7 39304265 PMC12254192

[B21] Fluck D. Fry C. H. Gulli G. Affley B. Robin J. Kakar P. . (2023). Adverse stroke outcomes amongst UK ethnic minorities: a multi-centre registry-based cohort study of acute stroke. Neurol. Sci. 44, 2071–2080. doi: 10.1007/s10072-023-06640-z 36723729 PMC9891657

[B22] Forster A. Ozer S. Crocker T. F. House A. Hewison J. Roberts E. . (2021). Longer-term health and social care strategies for stroke survivors and their carers: the LoTS2Care research programme including cluster feasibility RCT. Programme Grants Appl. Res. 9, 1–268. doi: 10.3310/pgfar09030 33819000

[B23] Frühwirth V. (2020). Apps in secondary prevention after stroke. Vienna Med. Weekly 170, 41–54. doi: 10.1007/s10354-019-00707-3 31535230

[B24] Fryer C. E. Luker J. A. McDonnell M. N. Hillier S. L. (2016). Self management programmes for quality of life in people with stroke. Cochrane Database Syst. Rev. 2016:CD010442. doi: 10.1002/14651858.CD010442.pub2 27545611 PMC6450423

[B25] Gomes F. Emery P. W. Weekes C. E. (2016). Risk of malnutrition is an independent predictor of mortality, length of hospital stay, and hospitalization costs in stroke patients. J. Stroke Cerebrovasc. Dis. 25, 799–806. doi: 10.1016/j.jstrokecerebrovasdis.2015.12.017 26796058

[B26] Gov.UK (2025). Fit for the future : 10 Year Health Plan for England. London: Gov.UK.

[B27] Groeneveld I. F. Arwert H. J. Goossens P. H. Vliet Vlieland T. P. M. (2018). The longer-term unmet needs after stroke questionnaire: cross-cultural adaptation, reliability, and concurrent validity in a Dutch population. J. Stroke Cerebrovasc. Dis. 27, 267–275. doi: 10.1016/j.jstrokecerebrovasdis.2017.08.043 28967592

[B28] Gurr H. Oliver L. Harvey O. Subedi M. Van Teijlingen E. (2024). The importance of positionality for qualitative researchers. Dhaulagiri J. Sociol. Anthropol. 48–54. doi: 10.3126/dsaj.v18i01.67553

[B29] Ha L. Hauge T. Spenning A. B. Iversen P. O. (2010). Individual, nutritional support prevents undernutrition, increases muscle strength and improves QoL among elderly at nutritional risk hospitalized for acute stroke: a randomized, controlled trial. Clin. Nutr. 29, 567–573. doi: 10.1016/j.clnu.2010.01.011 20176418

[B30] Hekmatpou D. Baghban E. M. Mardanian Dehkordi L. (2019). The effect of patient care education on burden of care and quality of life of caregivers of patients with stroke. J. Multidiscip. Healthc. 12, 211–217. doi: 10.2147/JMDH.S196903 30936715 PMC6430991

[B31] Holmes R. Ackerley S. Fisher R. J. Connell L. A. (2025). Exploring variation in the six-month review for stroke survivors: a national survey of current practice in England. BMC Health Serv. Res. 25:159. doi: 10.1186/s12913-025-12323-6 39871235 PMC11773788

[B32] Huppertz V. Guida S. Holdoway A. Strilciuc S. Baijens L. Schols J. M. G. A. . (2022). Impaired nutritional condition after stroke from the hyperacute to the chronic phase: a systematic review and meta-analysis. Front. Neurol. 12:780080. doi: 10.3389/fneur.2021.780080 35178021 PMC8846185

[B33] Intercollegiate Stroke Working Party (2023). National Clinical Guideline for Stroke for the UK and Ireland. London: Intercollegiate Stroke Working Party. Available online at: https://www.strokeguideline.org (accessed January 31, 2024).

[B34] Jaracz K. Grabowska-Fudala B. Jaracz J. Moczko J. Kleka P. Pawlicka A. . (2024). Caregiver burden after stroke: a 10-year follow-up study of Polish caregivers for stroke patients. BMC Nurs. 23:589. doi: 10.1186/s12912-024-02251-x 39183261 PMC11346017

[B35] Jones N. Mawson S. Drummond A. Booth L. O'Cathain A. (2025). The experiences of stroke survivors with eating and drinking difficulties, in acute stroke units: a qualitative inquiry. Disabil. Rehabil. 1–14. doi: 10.1080/09638288.2025.2563772 40985365

[B36] Jones N. Nasr N. (2018). The experiences of stroke survivors with managing eating 6 months post stroke. Br. J. Occup. Ther. 81, 106–115. doi: 10.1177/0308022617738487

[B37] Kalra L. Evans A. Perez I. Melbourn A. Patel A. Knapp M. . (2004). Training carers of stroke patients: randomised controlled trial. BMJ 328:1099. doi: 10.1136/bmj.328.7448.1099 15130977 PMC406319

[B38] Keller H. Payette H. Laporte M. Bernier P. Allard J. Duerksen D. . (2018). Patient-reported dietetic care post hospital for free-living patients: a Canadian Malnutrition Task Force Study. J. Hum. Nutr. Diet 31, 33–40. doi: 10.1111/jhn.12484 28524384

[B39] Kersten P. Low J. T. S. Ashburn A. George S. L. McLellan D. L. (2002). The unmet needs of young people who have had a stroke: results of a national UK survey. Disabil. Rehabil. 24, 860–866. doi: 10.1080/09638280210142167 12450462

[B40] King D. Wittenberg R. Patel A. Quayyum Z. Berdunov V. Knapp M. . (2020). The future incidence, prevalence and costs of stroke in the UK. Age Ageing 49, 277–282. doi: 10.1093/ageing/afz163 31957781 PMC7047821

[B41] Kronish I. M. Goldfinger J. Z. Negron R. Fei K. Tuhrim S. Arniella G. . (2014). Effect of peer education on stroke prevention. Stroke 45, 3330–3336. doi: 10.1161/STROKEAHA.114.006623 25248910 PMC4213208

[B42] Kruithof W. J. Post M. W. M. van Mierlo M. L. van den Bos G. A. M. de Man-van Ginkel J. M. Visser-Meily J. M. A. (2016). Caregiver burden and emotional problems in partners of stroke patients at two months and one year post-stroke: determinants and prediction. Patient Educ. Couns. 99, 1632–1640. doi: 10.1016/j.pec.2016.04.007 27103190

[B43] LoTS Care LUNS Study Team (2013). Validation of the longer-term unmet needs after stroke (LUNS) monitoring tool: a multicentre study. Clin. Rehabil. 27, 1020–1028. doi: 10.1177/0269215513487082 23787941

[B44] Malterud K. Siersma V. D. Guassora A. D. (2016). Sample size in qualitative interview studies. Qual. Health Res. 26, 1753–1760. doi: 10.1177/1049732315617444 26613970

[B45] McCullagh E. Brigstocke G. Donaldson N. Kalra L. (2005). Determinants of caregiving burden and quality of life in caregivers of stroke patients. Stroke 36, 2181–2186. doi: 10.1161/01.STR.0000181755.23914.53 16151029

[B46] McKevitt C. Fudge N. Redfern J. Sheldenkar A. Crichton S. Rudd A. R. . (2011). Self-reported long-term needs after stroke. Stroke 42, 1398–1403. doi: 10.1161/STROKEAHA.110.598839 21441153

[B47] Mooi N. M. Ncama B. P. (2020). Perceived needs of patients and family caregivers regarding home-based enteral nutritional therapy in South Africa: a qualitative study. PLoS One 15:e0228924. doi: 10.1371/journal.pone.0228924 32049983 PMC7015406

[B48] Morris S. Ramsay A. I. G. Boaden R. J. Hunter R. M. McKevitt C. Paley L. . (2019). Impact and sustainability of centralising acute stroke services in English metropolitan areas: retrospective analysis of hospital episode statistics and stroke national audit data. BMJ 364:l1. doi: 10.1136/bmj.l1 30674465 PMC6334718

[B49] Mou J. Sun J. Zhang R. Yang Y. Yang W. Zhao X. . (2022). Experiences and needs of home caregivers for enteral nutrition: a systematic review of qualitative research. Nurs. Open 9, 11–21. doi: 10.1002/nop2.990 34273248 PMC8685892

[B50] Nazari A. M. Abbaszadeh A. Kazemi R. Yousofvand V. Zandi M. (2024). The effect of online training based on stroke educational program on patient's quality of life and caregiver's care burden: a randomized controlled trial. BMC Nurs. 23:958. doi: 10.1186/s12912-024-02629-x 39736718 PMC11686964

[B51] Nehme A. Li L. (2026). The rising incidence of stroke in the young: epidemiology, causes and global impact. Int. J. Stroke 21, 14–23. doi: 10.1177/17474930251362583 40682212 PMC12743130

[B52] Ordovas J. M. Ferguson L. R. Tai E. S. Mathers J. C. (2018). Personalised nutrition and health. BMJ 361:k2173. doi: 10.1136/bmj.k2173 PMC6081996 29898881

[B53] Otsuki I. Himuro N. Tatsumi H. Mori M. Niiya Y. Kumeta Y. . (2020). Individualized nutritional treatment for acute stroke patients with malnutrition risk improves functional independence measurement: a randomized controlled trial. Geriatr. Gerontol. Int. 20, 176–182. doi: 10.1111/ggi.13854 31854054

[B54] Parappilly B. P. Mortenson W. B. Field T. S. Eng J. J. (2020). Exploring perceptions of stroke survivors and caregivers about secondary prevention: a longitudinal qualitative study. Disabil. Rehabil. 42, 2020–2026. doi: 10.1080/09638288.2018.1544296 30669873

[B55] Perry L. Hamilton S. Williams J. Jones S. (2013). Nursing interventions for improving nutritional status and outcomes of stroke patients: descriptive reviews of processes and outcomes. Worldviews Evid. Based Nurs. 10, 17–40. doi: 10.1111/j.1741-6787.2012.00255.x 22672585

[B56] Perry L. McLaren S. (2003). Eating difficulties after stroke. J Adv Nurs. 43, 360–369. doi: 10.1046/j.1365-2648.2003.02724.x 12887354

[B57] Pont W. Groeneveld I. Arwert H. Meesters J. Mishre R. R. Vliet Vlieland T. . (2020). Caregiver burden after stroke: changes over time? Disabil. Rehabil. 42, 360–367. doi: 10.1080/09638288.2018.1499047 30235954

[B58] Reeves M. J. Boden-Albala B. Cadilhac D. A. (2023). Care transition interventions to improve stroke outcomes: evidence gaps in underserved and minority populations. Stroke 54, 386–395. doi: 10.1161/STROKEAHA.122.039565 36689590

[B59] Rigby H. Gubitz G. Phillips S. (2009). A systematic review of caregiver burden following stroke. Int. J. Stroke 4, 285–292. doi: 10.1111/j.1747-4949.2009.00289.x 19689757

[B60] Rothwell K. Boaden R. Bamford D. Tyrrell P. J. (2013). Feasibility of assessing the needs of stroke patients after six months using the GM-SAT. Clin. Rehabil. 27, 264–271. doi: 10.1177/0269215512457403 22952306 PMC3652600

[B61] Sayer A. (2000). Realism and Social Science. London: SAGE Publications Ltd. doi: 10.4135/9781446218730

[B62] Scott D. (2005). Critical realism and empirical research methods in education. J. Philos. Educ. 39, 633–646. doi: 10.1111/j.1467-9752.2005.00460.x

[B63] Sentinel Stroke National Audit Programme (n.d.). Annual Results Portfolio for April 2024-March 2025. London: King's College London. Available online at: https://www.strokeaudit.org/results/Clinical-audit/National-Results.aspx (accessed September 15, 2025).

[B64] Shimazu S. Yoshimura Y. Kudo M. Nagano F. Bise T. Shiraishi A. . (2021). Frequent and personalized nutritional support leads to improved nutritional status, activities of daily living, and dysphagia after stroke. Nutrition 83:111091. doi: 10.1016/j.nut.2020.111091 33388653

[B65] Smart A. Harrison E. (2017). The under-representation of minority ethnic groups in UK medical research. Ethn. Health 22, 65–82. doi: 10.1080/13557858.2016.1182126 27174778

[B66] The Stroke Association (2018). The Stroke Association Lived experience of Stroke Survivors. London: The Stroke Association.

[B67] Tsai P. C. Yip P. K. Tai J. J. Lou M. F. (2015). Needs of family caregivers of stroke patients: a longitudinal study of caregivers' perspectives. Patient Prefer. Adherence 9, 449–457. doi: 10.2147/PPA.S77713 25834409 PMC4370911

[B68] Vanwinkelen K. Spruyt B. Smits T. (2026). Digital interventions targeting healthy and sustainable eating behavior: systematic review and meta-analysis. J. Med. Internet Res. 28:e80821. doi: 10.2196/80821 41505650 PMC12782463

[B69] Zawawi N. S. M. Aziz N. A. Fisher R. Ahmad K. Walker M. F. (2020). The Unmet needs of stroke survivors and stroke caregivers: a systematic narrative review. J. Stroke Cerebrovasc. Dis. 29:104875. doi: 10.1016/j.jstrokecerebrovasdis.2020.104875 32689648

[B70] Zhao Y. Xu Y. Ma D. Fang S. Zhi S. He M. . (2024). The impact of education/training on nurses caring for patients with stroke: a scoping review. BMC Nurs. 23:90. doi: 10.1186/s12912-024-01754-x 38308293 PMC10835862

