# Supplementary material for: Stroke survivors' and carers' experiences of nutritional care after stroke: a qualitative study
Source: Front Stroke. 2026 May 8;5:1733430. doi: 10.3389/fstro.2026.1733430 (PMC13193854; doi:10.3389/fstro.2026.1733430)
Supplement: Supplementary file 1 [file Data_Sheet_1.docx]

**Stroke survivor and caregiver experiences of nutritional care during stroke recovery**

Interview Guide for Researchers

**Research Aim:** to explore experiences of nutritional care in the setting of stroke recovery from the perspectives of stroke survivors and caregivers.

**Introduction:**

We wish to conduct a series of semi-structured interviews with stroke survivors and their caregivers, to gain a better understanding of what are the key issues to do with nutritional care following a stroke, the barriers and enablers in helping stroke survivors meet their nutritional needs along the care pathway, what changes they have made, support they have accessed and what additional help they feel should be available to stroke survivors. We would like to ask some questions about this and ask you to give us your views. All information provided will be treated confidentially and used solely for the purpose of this research project.

**Interview:**

Start with welcome and introductions. Scene setting and ensuring the participant is comfortable and consents to taking part in the study. Make it clear that the participant can ask to take a break, or for the interview to stop at any point.

Demographic information will be collected at this stage.

Stroke survivor questions:

1. What was your experience of eating and drinking following your stroke:
   1. In hospital
   2. On discharge from hospital
   3. In the first 6 months
   4. More recently
2. What nutritional goals have you had since your stroke?
   1. Did your weight change following the stroke?
   2. How did the stroke impact your appetite?
   3. How did the stroke impact your energy levels?
3. What barriers to meeting nutritional goals did you identify and how did you overcome them?
4. What facilitators to meeting nutritional goals did you identify?
5. What nutritional education and support did you receive, and what would you have liked?
6. How do you perceive the role of nutrition in rehabilitation and recovery from a stroke, and how has this view changed over time?
7. How would you describe your experience of nutritional interventions, such as food fortification, oral nutritional supplements and other dietary interventions, such as vitamin and minerals? omega-3, probiotics, antioxidants etc. If they have not used or considered, then the interviewers will explore the reasons behind this.
8. What changes have you made to your diet since the stroke and has this had any noticeable impact? e.g. to body composition, physical performance, mood or cognition.
9. How confident do you feel with regards what to eat and drink to help prevent another stroke?
10. How do you think your nutritional care could have been improved?

Carer questions:

With regards the person to whom you have been providing care to following their stroke:

1. What was your experience of their eating and drinking following the stroke:
   1. In hospital
   2. On discharge from hospital
   3. In the first 6 months
   4. More recently
2. What nutritional goals have they had since their stroke?
   1. How did their weight change following the stroke?
   2. How did the stroke impact their appetite?
   3. How did the stroke impact their muscle or energy levels
3. What barriers to meeting their nutritional goals did you identify and how did you overcome them?
4. What facilitators to meeting their nutritional goals did you identify?
5. What nutritional education and support did you receive, and what would you have liked?
6. How has the stroke affected you as a caregiver? Has it impacted your eating and drinking?
7. How do you perceive the role of nutrition in rehabilitation and recovery from a stroke, and how has this view changed over time? Do they have any other conditions e.g. diabetes, constipation?
8. How would you describe your experience of nutritional interventions, such as food fortification, oral nutritional supplements and other dietary interventions, such as vitamin and minerals? omega-3, antioxidants, pre or probiotics, cholesterol drinks etc. If they have not used or considered, then the interviewers will explore the reasons behind this.
9. What changes have you helped them make to their diet since the stroke and has this had any noticeable impact? e.g. to body composition, physical performance, mood or cognition.
10. How confident do you feel with regards to knowing what they should eat and drink to help prevent another stroke?
11. How do you think their nutritional care could have been improved?

Next steps:

- Thank the participant.
- Ask whether they have any remaining questions about the research.
- Reassure them about confidentiality and pseudo-anonymity.
- Explain the process for payment.
